# Uropathogen antibiogram regional variations—Are Australian antimicrobial guidelines appropriate?

**DOI:** 10.1002/bco2.429

**Published:** 2024-09-15

**Authors:** Gavin Wei, James Antony Sidney Sewell, Caroline Bartolo, Amelia Pearce, Owen Harris, Richard Grills

**Affiliations:** ^1^ Department of Urology Monash Health Melbourne Victoria Australia; ^2^ Department of Surgery, School of Clinical Sciences Monash University Melbourne Victoria Australia; ^3^ Department of Urological Surgery, Barwon Health University Hospital Geelong Geelong Victoria Australia; ^4^ Department of Infectious Diseases, Barwon Health University Hospital Geelong Geelong Victoria Australia; ^5^ Department of Microbiology, Geelong Laboratory Australian Clinical Laboratories Geelong Victoria Australia; ^6^ Department of Surgery, School of Medicine Deakin University Geelong Victoria Australia

**Keywords:** antibiogram, antibiotics, resistance, urinary tract infections, uropathogen, variation

## Abstract

**Objectives:**

The objectives of this study are as follows: to assess the uropathogen antibiogram at two tertiary hospitals in Victoria to look at the difference in susceptibility patterns, to assess whether national guideline recommendations were applicable and to assess the feasibility of local antibiogram analysis to guide development of hospital‐specific guidelines for empirical treatment of urosepsis and for pre‐operative prophylaxis for urological procedures.

**Patients and methods:**

All positive urine cultures analysed at Barwon Health and Monash Health, two tertiary hospitals in regional and metropolitan Victoria, Australia, respectively, between January 2019 and December 2020 were retrospectively identified. Data obtained included the organism cultured and their susceptibility profiles.

**Results:**

Three thousand seven hundred and seventy‐seven positive urine cultures from Barwon Health and 6821 from Monash Health were identified. The most common uropathogen was *Escherichia coli* , which was cultured in 53.4% and 59.1% of urine cultures at Barwon Health and Monash Health, respectively. The main differences observed were in *Enterococcus* spp., which were cultured in 8.8% and 4.9% of cultures at Barwon Health and Monash Health, respectively, and *Candida* spp. in 4.2% and 1.5% of cultures at Barwon Health and Monash Health, respectively. The largest differences were found in fluoroquinolone resistance with 12.1% of organisms resistant to ciprofloxacin at Barwon Health compared to 6.4% at Monash Health and 7.1% of organisms resistant to vancomycin compared to 20.1% at Barwon Health and Monash Health, respectively.

**Conclusion:**

This study demonstrates that there is considerable variability in the uropathogens and their antimicrobial susceptibility profile in two large health services in the same state. We recommend that each centre performs regular analysis of their uropathogen antibiogram to develop local guidelines for treatment and pre‐operative prophylaxis for uropathogens.

## 1 INTRODUCTION

Urinary tract infections are an important cause of hospitalisation and post‐operative infection worldwide.^1^ The severity can range from uncomplicated cystitis to life‐threatening sepsis. Owing to overuse and misuse of antimicrobial agents, there has been a global increase in antimicrobial resistance.^2^ The degree of resistance has been found to have significant geographic variability due to local antimicrobial guidelines and practices.^3^, ^4^, ^5^, ^6^ Consequently, the current Australian Therapeutic Guidelines recommend that antibiotic regimens may need to be modified according to patient factors and environmental factors, including organisms causing infection within the institution and their susceptibilities.^7^ Similarly, the current European Association of Urology (EAU) guidelines suggest that empirical antimicrobial treatment should be tailored to local pathogen prevalence, susceptibility profiles and virulence.^8^

Current Australian guidelines recommend 4–5 mg/kg of intravenous gentamicin together with 2 g of intravenous ampicillin or amoxicillin 6‐hourly as empirical treatment for urosepsis.^9^ In instances where gentamicin is contraindicated, 1 g ceftriaxone every 12 h or 2 g cefotaxime 8‐hourly is recommended.^9^ Pre‐procedural antimicrobial prophylaxis has been widely adopted prior to select urological procedures as a strategy to minimise the rates of post‐operative infections.^10^ Current guidelines within Australia recommend pre‐operative screening for bacteriuria in elective urological procedures that enter the urinary tract aside from uncomplicated cystoscopic procedures for diagnostic purposes alone.^10^ For prophylactic purposes, the use of a single dose of 2 mg/kg of intravenous gentamicin within 120 minutes of the procedure or, if gentamicin is contraindicated, 2 g of intravenous cefazolin within 60 minutes of procedure is recommended.^10^

Sepsis from a urinary tract source is associated with high mortality. Therefore, knowledge of the local uropathogen antibiogram is crucial for clinicians when choosing empiric antimicrobial therapy. Although local antibiograms are commonly available through the local microbiology department, they may not be freely available for review by prescribing units. In this current study, we aimed to assess the local uropathogens and their antimicrobial resistance profile at two hospitals within Victoria, Australia, to evaluate the suitability of national guidelines and compare this with other domestic and international centres. By doing so, we aim to highlight the importance of local antibiograms in a urological context.

## 2 PATIENTS AND METHODS

A retrospective observational study was conducted to assess the local uropathogen population and their antimicrobial resistance profiles. All positive urine cultures during a 24‐month period between January 2019 and December 2020 from Barwon Health and Monash Health, two large tertiary hospitals in Victoria, were retrospectively identified. Inpatient and outpatient samples were included as part of a convenience sampling approach. Ethical approval for this project was sought and granted by each institution's Human Research Ethics Committee as part of Quality Assurance (Barwon Health reference 19/50; Monash Health reference RES‐21‐0000‐369Q).

Urine samples were analysed and processed based on local microbiology laboratory standards. Both microbiology labs performed antimicrobial sensitivity testing and interpretation according to CLSI guidelines. The majority of organisms underwent automated sensitivity testing using Vitek. For organisms that were unable to be tested using Vitek, manual sensitivity via disc diffusion was utilised. Cultured organisms were not tested for sensitivity to all antibiotics; if the organism had known intrinsic resistance to an antibiotic class (such as *Enterococcus* spp. to cephalosporins), then these antibiotics were not included in the susceptibility testing.

Data obtained included the organism cultured and susceptibility profiles although resistance mechanisms were not assessed. During data analysis, organisms were divided into *Escherichia coli* (*E. coli*), *Enterococcus* spp., *Klebsiella* spp., *Pseudomonas* spp., *Proteus* spp., *Streptococcus* spp., *Staphylococcus* spp., *Candida* spp. and other organisms. Antimicrobial resistance rates were compiled for the pathogens overall and for each bacterial subgroup. Resistance rate calculations only included cultures where the antimicrobial agent was tested for. Data analysis was conducted using Microsoft Excel.

## 3 RESULTS

Over the specified time period, there were 3777 positive urine cultures from Barwon Health and 6821 from Monash Health. The most common uropathogen was *E. coli*, which was cultured in 53.4% and 59.1% of urine cultures at Barwon Health and Monash Health, respectively. *Enterococcus* spp. were cultured in 8.8% and 4.9% of cultures, *Klebsiella* spp. in 10.2% and 11.3% of cultures, *Pseudomonas* spp. in 5.6% and 5.3% of cultures, *Proteus* spp. in 4.9% and 5.6% of cultures, *Streptococcus* spp. in 3.0% and 0.9% of cultures, *Staphylococcus* spp. in 3.5% and 4.2% of cultures and *Candida* spp. in 4.2% and 1.5% of cultures at Barwon Health and Monash Health, respectively. Various other species were identified in 6.4% and 7.1% of cultures at Barwon Health and Monash Health, respectively. The distribution of each organism subgroup is expressed in Table 1 and compared with similar international studies.

**TABLE 1:** Uropathogen microbiome distribution across domestic and international hospitals.

	Barwon Health (*n* = 3777)	Monash Health (*n* = 6821)	India^3^ (*n* = 661)	Mexico^4^ (*n* = 1512)	France^6^ (*n* = 1160)	Cameroon^5^ (*n* = 55)
*Escherichia coli*	53.4%	59.1%	61.4%	67.3%	74.6%	50.9%
*Enterococcus* species	8.8%	4.9%	0.6%	6.0%	5.4%	Not reported
*Klebsiella* species	10.2%	11.3%	14.2%	6.4%	3.4%	16.4%
*Pseudomonas* species	5.6%	5.3%	2.1%	7.1%	Not reported	1.8%
*Proteus* species	4.9%	5.6%	8.0%	3.1%	5.9%	9.1%
*Streptococcus* species	3.0%	0.9%	Not reported	0.5%	2.2%	Not reported
*Staphylococcus* species	3.5%	4.2%	2.6%	2.0%	3.5%	3.6%
*Candida* species	4.2%	1.5%	8.3%	3.4%	Not reported	Not reported
Other	6.4%	7.1%	2.8%	4.2%	5.0%	18.2%

Resistance rates at the hospitals varied. In the following paragraph, Barwon Health resistance rates are listed first followed by Monash Health resistance rates. The largest differences were found in fluoroquinolone resistance (ciprofloxacin 12.1%/16.4% and norfloxacin: 7.1%/20.6%), and in vancomycin (7.1%/20.1%). Other antibiotic resistance rates were more similar. The resistance rates for penicillins were ampicillin (51.3%/62.4%), amoxicillin‐clavulanic acid (19.3%/19.9%) and piperacillin‐tazobactam (8.1%/6.5%). The resistance rates for first generation cephalosporins were cefalexin (30.3%/21.5%) and cefazolin (37.9%/30.3%). The resistance rates for third generation cephalosporins were ceftazidime (20.9%/14.1%) and ceftriaxone (24.8%/19.1%). A total of 17.3%/12.8% of organisms were resistant to cefepime; 0.6%/0.4% of organisms were resistant to meropenem; 5.4%/6.7% of organisms were resistant to gentamicin. The resistance rates for antifolate antimicrobials were trimethoprim (24.4%/30.9%) and cotrimoxazole (23.2%/27.5%). A total of 17.9%/11.7% of organisms were resistant to nitrofurantoin. These results are summarised in Table 2 along with resistance rates for each organism subgroup.

**TABLE 2:** Antimicrobial resistance rates of micro‐organisms, categorised by micro‐organism subgroup.

	Overall		*Escherichia coli*		*Enterococcus* species		*Klebsiella* species		*Pseudomonas* species		*Proteus* species		*Streptococcus* species		*Staphylococcus* species		Other	
	BH	MH	BH	MH	BH	MH	BH	MH	BH	MH	BH	MH	BH	MH	BH	MH	BH	MH
AMP	51.3	62.4	46.9	53	16.6	61.9	x	x	x	x	23.2	27.3	0.9	17.7	77.7	15.8	94.1	98.7
AUG	19.3	19.9	14.1	8	x	x	15.5	14.7	x	x	9.2	12.5	x	x	21.1	x	81.7	77.4
PIT	8.1	6.5	5.5	4	x	x	14.6	10.5	2.1	19.7	0.0	1.9	x	x	x	x	23.4	16.1
CEX	30.3	21.5	23.8	16	x	x	27.8	21.7	x	x	10.0	14.2	0.0	x	x	50.0	88.9	78.7
CFZ	37.9	30.3	30.8	20	x	x	39.7	29.7	x	x	13.3	24.5	x	x	20.8	x	89.4	79.4
CAZ	20.9	14.1	26.6	15	x	x	13.9	9.8	2.9	14.5	8.2	6.8	x	x	x	x	27.7	19.1
CRO	24.8	19.1	26.7	15	x	x	15.7	10.0	x	x	8.2	8.1	x	7.0	33.3	x	32.0	21.1
FEP	17.3	12.8	26.3	15	x	x	7.0	7.7	2.6	13.6	5.9	5.9	x	x	x	x	7.0	6.7
MEM	0.6	0.4	0.1	0	x	x	0.0	0.6	2.1	6.1	1.2	0	x	x	x	x	1.4	0.0
GEN	5.4	6.7	6.9	8	x	x	1.6	2.0	2.4	3.9	3.1	6.5	x	x	2.1	10.6	4.8	4.1
TRI	24.4	30.9	27.4	32	x	x	10.6	13.5	x	x	23.8	27.9	x	x	0.0	18.5	15.7	12.4
COT	23.2	27.5	32.3	29	x	x	10.3	8.7	x	x	10.5	20.3	11.1	0	6.2	13.8	10.6	6.3
NIT	17.6	11.7	2.5	1	x	x	43.7	0.0	x	x	100.0	100	0.0	x	0.0	0	54.7	97.1
NOR	7.1	20.6	8.2	25	x	x	1.8	11.1	3.4	0	1.1	9.3	x	x	11.6	x	3.2	8.0
CIP	12.1	16.4	17.2	16	x	x	1.8	5.4	4.1	11.2	4.8	9.3	x	x	11.7	86.8	4.9	6.0
VAN	7.1	20.1	x	x	9.4	37.7	x	x	8.3	x	5.9	x	6.7	0	0.0	0	4.9	0.0

*Note*: All results expressed as percentage resistant.

Abbreviations: AMP, ampicillin; AUG, amoxicillin‐clavulanic acid; BH, Barwon Health; CAZ, ceftazidime; CEX, cefalexin; CFZ, cefazolin; CIP, ciprofloxacin; COT, cotrimoxazole; CRO, ceftriaxone; FEP, cefepime; GEN, gentamicin; MEM, meropenem; MH, Monash Health; NIT, nitrofurantoin; NOR, norfloxacin; PIT, piperacillin‐tazobactam; TRI, trimethoprim; VAN, vancomycin; x, not tested for.

## 4 DISCUSSION

The utilisation of appropriate empirical antibiotics in urosepsis as well as in pre‐procedural prophylaxis for select urological procedures has been recommended in major urological guidelines.^8^, ^11^, ^12^ With the increase in antimicrobial resistance worldwide, these guidelines have suggested that the choice of empirical therapy should consider the local epidemiology of uropathogens and their antimicrobial resistance profile. Our study compares uropathogens and their antimicrobial susceptibility profile between health services within close proximity and demonstrates the necessity for urology and infectious diseases departments to cooperate and create local prescribing guidelines.

There are considerable differences between domestic and international centres in the distribution of uropathogens. In our attempt to assess local variations, we analysed two health services in Victoria, Australia. Monash Health is a metropolitan hospital in suburban Melbourne whilst Barwon Health is a hospital approximately 100 km away in the regional centre of Geelong.

*E. coli* was the most commonly isolated organism, and prevalence was slightly higher at Monash Health compared to Barwon Health, whereas there were more *Enterococcus* spp., *Streptococcus* spp. and *Candida* spp. isolated at Barwon Health. This difference could potentially be explained by a difference in patient demographics. Amongst the *Enterococcus* spp., there was a higher proportion of *Enterococcus faecalis* (82.8%) at Barwon Health compared to Monash Health (37.1%) whereas *Enterococcus faecium* was more prevalent in Monash Health (62.2% compared to 8.1%). This explains the difference in vancomycin susceptibility between the two health services. In comparison to international cohorts, a higher proportion of *E. coli* (61.42%), *Klebsiella* spp. (14.22%), *Pseudomonas* spp. (8.02%) and *Candida* spp. (8.32%) but fewer *Proteus* spp. (2.12%) and *Enterococcus* spp. (0.61%) were identified in an Indian tertiary centre.^3^ In a Mexican tertiary centre, a higher prevalence of *E. coli* (67.28%) and *Pseudomonas* spp. (7.12%) were found along with fewer *Klebsiella* spp. (6.43%) and *Proteus* spp. (3.08%).^4^ Whilst these studies demonstrated that regional variation exists between countries, in our study, we identified variations in the uropathogens even between health services in close proximity that may affect the selection of empirical treatment for uropathogens. These variations emphasise the importance of local antibiogram analysis and collaboration between urology and infectious diseases departments in order to create institution specific guidelines to guide empirical antibiotic choice for urosepsis and pre‐procedural prophylaxis.

The antimicrobial resistance profile also differed between local centres. Overall, there were higher rates of resistance within Barwon Health to first, third and fourth generation cephalosporins suggesting higher rates of extended spectrum beta‐lactamase (ESBL) organisms compared to Monash Health although resistance mechanisms were not assessed in this study. Contrastingly, there were higher rates of vancomycin‐resistant *Enterococcus* spp. (VRE) at Monash Health with 37.7% resistant compared to 9.4% at Barwon Health. Compared to other Australian studies assessing *E. coli* sensitivities in Western Australia (WA) in 2019, the Northern Territory (NT) in 2019 and Australian Capital Territory (ACT) in 2013, there were varying rates of resistance to amoxicillin‐clavulanic acid (14.1% at Barwon Health, 8% at Monash Health, 16.3% in WA, 5.8% in NT and 9.0% in ACT) and similar rates of resistance to gentamicin (6.9% at Barwon Health, 8% at Monash Health, 8.2% in NT and 4.9% in ACT) at our centres.^13^, ^14^ However, in our centres, there were higher rates of resistance to cefazolin (30.8% at Barwon Health, 20% at Monash Health, 9.6% in WA, 14.2% in NT and 12.8% in ACT), extended spectrum cephalosporins (26% at Barwon Health, 15% at Monash Health, 5.3% in WA and 8.2% in NT) and fluoroquinolones (17.2% at Barwon Health, 16% at Monash Health, 11.3% in WA, 10.8% in NT, 10.1% in ACT). Compared to international studies, a Mexican study reported higher rates of resistance to amoxicillin‐clavulanic acid (72.4%), cefazolin (53.5%), extended spectrum cephalosporins (47.6%–49.3%), meropenem (7.4%), gentamicin (47.5%), cotrimoxazole (62.9%) and ciprofloxacin (72.9%).^4^ A French study reported higher rates of resistance to amoxicillin‐clavulanic acid (36.3%) but lower rates of resistance to gentamicin (2.5%) and ciprofloxacin (3.2%).^6^ It is recognised that these differences are a reflection of local antimicrobial practices.^15^, ^16^ These studies demonstrate that there is considerable variability in the antimicrobial resistance profiles even between local centres, meaning that national guidelines may not necessarily provide the optimal approach for decisions about empirical antimicrobials at a local level. Therefore, local urology and infectious diseases teams should be encouraged to work together to utilise local antibiograms to create guidelines that improve antimicrobial stewardship.

Current Australian empirical antimicrobial recommendations of gentamicin provide adequate coverage for urosepsis and pre‐operative prophylaxis at both studied centres. In our study, most organisms were susceptible to gentamicin with 5.4% and 6.7% being resistant at Barwon Health and Monash Health, respectively. However, owing to nephrotoxicity, vestibular and auditory toxicity, alternatives to gentamicin are sometimes preferred. In situations where gentamicin is contraindicated, the antibiotic recommendations may be less efficacious to treat organisms at our centres. For urosepsis, ceftriaxone was recommended in cases where gentamicin was contraindicated. Ceftriaxone resistance was present in 24.8% of positive cultures at Barwon Health and 19.1% of positive cultures at Monash Health—and note that overall susceptibility rate of an unselected infection would be lower, as due to intrinsic cephalosporin resistance in *Enterococcus* spp. and *Candida* spp., these were not tested. However, for patients with severe sepsis, gentamicin may be added, and vancomycin is recommended in addition if there has been recent instrumentation. Although options such as piperacillin‐tazobactam would give broader empiric cover of reported organisms, this must be balanced against the risk of increasing antimicrobial resistance. For surgical prophylaxis, cefazolin is recommended if gentamicin was contraindicated. At Barwon Health, however, amoxicillin‐clavulanic acid is recommended prior to most procedures. In our cohorts, we found up to 37.9% and 30.3% of organisms were resistant to cefazolin whereas amoxicillin‐clavulanic acid had resistance rates of 14.1% and 8% at Barwon Health and Monash Health, respectively. Whilst pre‐procedural prophylaxis can be guided by pre‐operative urine culture and sensitivities, our recommendation is, in cases where gentamicin was contraindicated, for higher risk procedures such as transurethral resection of the prostate in a catheter‐dependent patient or percutaneous nephrolithotomy, broader coverage can be considered in collaboration with the local infectious diseases and antimicrobial stewardship team. These discrepancies between the antibiograms observed in our cohorts and the national guidelines highlight the importance of clinicians being cognisant of the local antimicrobial susceptibility profile of urine culture organisms to guide empirical treatment choices rather than relying purely on country or international society‐based guidelines.

Beyond the generation of traditional antibiograms, other variations on antibiograms have been described to improve empiric antibiotic choice. The weighted‐incidence syndromic combination antibiogram (WISCA) is a method that aims to estimate the likelihood that an antibiotic regimen would treat all relevant organisms for a specific infection syndrome.^17^ Furthermore, the WISCA can be stratified by patient factors such as age, comorbidities and whether they are from a nursing home.^17^ This enables more targeted empiric antibiotic treatment recommendations and has been utilised in specific populations such as paediatric UTIs.^18^ Therefore, there is potential for urology and infectious diseases teams to explore how WISCAs can be generated for urology specific presentations such as infected, obstructed systems or patients undergoing procedures with higher risk of post‐operative urosepsis to enable improved antibiotic choices in these settings.

This study was limited by the inability to further stratify the database by urine cultures collected in a community or hospital setting. As community isolates tend to be more susceptible overall to antimicrobial agents than hospital isolates, collating both of these results may reflect a sampling bias and lead to an underestimation of the true resistance rate for patients who require hospital admission with urosepsis.^14^ Furthermore, this study was unable to stratify the database to only include patients undergoing urological procedures. Whilst we acknowledge that some urology patients may have risk factors for multi‐drug resistant urinary tract infections such as long‐term bladder catheterisation and urinary incontinence,^19^ these patients often have historical or pre‐procedural urine culture results to help guide prophylaxis. Consequently, studying the broader uropathogen antibiogram provides an appropriate representation of most patients undergoing urological procedures without pre‐operative urine culture. This study was further limited by slight intrinsic differences between the two laboratories in susceptibility testing. Additionally, some samples may have been taken from urinary catheters thus representing colonisation rather than true infection. Importantly, clinical outcomes were not assessed in this study.

In conclusion, this study demonstrates that there is considerable variability in the uropathogen and antimicrobial susceptibility profile, even between local centres. For our cohorts, the national therapeutic guidelines recommended some antimicrobials to which our uropathogens demonstrated high levels of resistance at both centres. This information has allowed the relevant hospitals to develop local guidelines that better suit their antibiograms for urinary culture results. We recommend that each centre should perform regular analysis of their local urine culture results and susceptibility profiles and work with their microbiology laboratory and antimicrobial stewardship team to develop local guidelines for pre‐operative prophylaxis. Future studies may aim to look at specific populations such as urology specific presentations or procedures at higher risk of post‐operative infection. Additionally, the clinical significance of these differences may be assessed by auditing antimicrobial choice for surgical prophylaxis and comparing the rates of post‐operative infection. Similarly, empirical antimicrobial choices for urinary sepsis can be audited, assessing the clinical outcomes of urinary sepsis. These studies may help inform changes to local guidelines. Furthermore, individual approaches to antibiotic selection that consider risk factors and clinical condition can help guide antibiotic choice and potentially reduce the risk of antimicrobial resistance.

## AUTHOR CONTRIBUTIONS

**Gavin Wei:** Data curation; writing—original draft; methodology. **James Antony Sidney Sewell:** Data curation; writing—original draft; conceptualisation; methodology. **Caroline Bartolo:** Writing—review and editing. **Amelia Pearce:** Data curation. **Owen Harris:** Writing—review and editing. **Richard Grills:** Conceptualisation; methodology; writing—review and editing; supervision.

## CONFLICT OF INTEREST STATEMENT

The authors have no relevant financial or non‐financial interests to disclose.
